# Delineating morbillivirus entry, dissemination and airborne transmission by studying *in vivo* competition of multicolor canine distemper viruses in ferrets

**DOI:** 10.1371/journal.ppat.1006371

**Published:** 2017-05-08

**Authors:** Rory D. de Vries, Martin Ludlow, Alwin de Jong, Linda J. Rennick, R. Joyce Verburgh, Geert van Amerongen, Debby van Riel, Peter R. W. A. van Run, Sander Herfst, Thijs Kuiken, Ron A. M. Fouchier, Albert D. M. E. Osterhaus, Rik L. de Swart, W. Paul Duprex

**Affiliations:** 1 Department of Viroscience, Postgraduate School of Molecular Medicine, Erasmus MC, Rotterdam, The Netherlands; 2 Department of Microbiology, Boston University School of Medicine, Boston, Massachusetts, United States of America; Friedrich-Loeffler-Institut, GERMANY

## Abstract

Identification of cellular receptors and characterization of viral tropism in animal models have vastly improved our understanding of morbillivirus pathogenesis. However, specific aspects of viral entry, dissemination and transmission remain difficult to recapitulate in animal models. Here, we used three virologically identical but phenotypically distinct recombinant (r) canine distemper viruses (CDV) expressing different fluorescent reporter proteins for *in vivo* competition and airborne transmission studies in ferrets (*Mustela putorius furo*). Six donor ferrets simultaneously received three rCDVs expressing green, red or blue fluorescent proteins via conjunctival (ocular, Oc), intra-nasal (IN) or intra-tracheal (IT) inoculation. Two days post-inoculation sentinel ferrets were placed in physically separated adjacent cages to assess airborne transmission. All donor ferrets developed lymphopenia, fever and lethargy, showed progressively increasing systemic viral loads and were euthanized 14 to 16 days post-inoculation. Systemic replication of virus inoculated via the Oc, IN and IT routes was detected in 2/6, 5/6 and 6/6 ferrets, respectively. In five donor ferrets the IT delivered virus dominated, although replication of two or three different viruses was detected in 5/6 animals. Single lymphocytes expressing multiple fluorescent proteins were abundant in peripheral blood and lymphoid tissues, demonstrating the occurrence of double and triple virus infections. Transmission occurred efficiently and all recipient ferrets showed evidence of infection between 18 and 22 days post-inoculation of the donor ferrets. In all cases, airborne transmission resulted in replication of a single-colored virus, which was the dominant virus in the donor ferret. This study demonstrates that morbilliviruses can use multiple entry routes in parallel, and co-infection of cells during viral dissemination in the host is common. Airborne transmission was efficient, although transmission of viruses expressing a single color suggested a bottleneck event. The identity of the transmitted virus was not determined by the site of inoculation but by the viral dominance during dissemination.

## Author summary

Canine distemper virus (CDV) infection of ferrets is a tractable animal model for measles. Ferrets are highly susceptible to CDV, and inoculation with a low dose leads to lethal disease. We performed *in vivo* competition experiments to study virus entry, dissemination and transmission. Ferrets were simultaneously inoculated with CDV via the conjunctival, intra-nasal and intra-tracheal routes. The viruses were identical except for the fluorescent reporter protein encoded by the viral genome. By detecting cells expressing the different fluorescent reporter proteins at various sites in the host, we determined that CDV can enter the host in parallel at multiple sites. Virus spread in the ferret occurred via infected lymphocytes, which often turned out to be double- or triple-infected. Sentinel ferrets, placed in physically separated adjacent cages, became infected by airborne transmission. Transmission of the dominant single color despite replication of multicolor viruses in the upper respiratory tract suggested a bottleneck event.

## Introduction

Morbilliviruses are enveloped, non-segmented, negative strand RNA viruses that belong to the family *Paramyxoviridae* [1]. They are highly contagious, spread via the respiratory route, cause profound immune suppression but also elicit lifelong immunity in surviving hosts, and have a propensity to cause large outbreaks associated with high morbidity and mortality in previously unexposed populations. Measles virus (MV) is the prototype morbillivirus and remains a significant cause of childhood morbidity and mortality in the developing world. Measles is characterized by fever, skin rash, cough and conjunctivitis, followed by a transient immune suppression [2]. The resulting increased susceptibility to secondary infections can lead to life-threatening complications [3]. In spite of the availability of safe and effective live-attenuated MV vaccines, measles outbreaks continue to occur in the industrialized world due to inadequate vaccination coverage and importations of this highly transmissible virus from endemic regions [4, 5].

The narrow host range and long incubation period of MV have restricted the characterization of its pathogenesis since patients are not recognized as having measles until onset of rash, and animal studies predominantly rely on experimental infections of non-human primates (NHPs). A surrogate model for MV pathogenesis is infection of ferrets with canine distemper virus (CDV), a morbillivirus that can infect a wide range of carnivores [6, 7]. However, CDV in carnivores is highly neurotropic and often leads to fatal disease [8–12], which is in sharp contrast to MV infection of humans and NHPs.

Morbilliviruses are amongst the most contagious viruses known and are primarily transmitted by aerosols or respiratory droplets. Once inhaled, virions establish primary infection by receptor-dependent fusion at the plasma membrane [13]. Two cellular receptors involved in morbillivirus infection have been identified: signaling lymphocyte activation molecule family member 1 (SLAM/F1, or CD150), expressed by subsets of thymocytes, dendritic cells (DCs), hematopoietic stem cells, macrophages, T- and B-lymphocytes [14], and nectin cell adhesion molecule 4 (nectin-4, previously known as poliovirus receptor-related 4), expressed at the adherens junction complex of epithelial cells [15, 16]. Both receptors play a crucial role in viral pathogenesis (reviewed in [17]), with CD150-mediated infection being critical for entry and dissemination [18, 19] and nectin-4-mediated infection critical for virus transmission [20, 21].

A number of aspects of morbillivirus pathogenesis remain unresolved. Studies in mice and NHPs have shown that MV initially infects alveolar macrophages and DCs in the lungs, instead of epithelial cells of the upper respiratory tract [22–24]. Even though this is a possible entry route, it seems unlikely that a highly contagious virus with an R_0_ of 12–18 [25] and of which infection with one 50% tissue culture infectious dose (TCID_50_) is sufficient to cause productive infection in macaques [26], exclusively depends on infection of target cells in the alveoli. Additional routes of entry into a susceptible host have been postulated, including infection of CD150^+^ immune cells within the epithelium of the respiratory tract [18, 27]. As DCs are abundant within this epithelium, and DC-SIGN was previously identified as an attachment receptor for MV [28, 29], it is suggested that DCs could play a crucial role in this entry route [30]. Additionally, damage to the upper respiratory tract epithelium by mechanical injury [31] or respiratory co-infections potentially exposes nectin-4 as a cellular entry receptor. Another potential route of entry for morbilliviruses involves CD150^+^ and/or DC-SIGN^+^ cells present in the conjunctiva of the eye [32, 33]. However, direct *in vivo* evidence from animal models for these alternative entry strategies is lacking.

Recombinant (r) viruses expressing fluorescent reporter proteins have allowed sensitive assessment of morbillivirus entry and dissemination *in vivo* [18, 34, 35]. Multicolored viruses have been used to demonstrate MV polyploidy [36], ribonucleoprotein trafficking [37], superinfection immunity [38], potential of segmenting the genome [39] and a cross-genomic cooperation modulating phenotype [40]. Recognizing the power of this approach for *in vivo* competition studies, we simultaneously administered three rCDVs expressing green (Venus), red (dTom) or blue (TagBFP) fluorescent proteins to ferrets to assess viral entry, intra-host dissemination and inter-host transmission. We show that CDV enters the host efficiently if delivered to the nose or lung, and that infection of the host through conjunctival administration, although less efficient, is also possible. *In vivo* competition showed that the route of entry had no influence on viral dissemination. However, exclusive single-color virus transmission of the systemically dominant virus to recipient ferrets was observed, suggesting a bottleneck during transmission.

## Results

### Recombinant CDVs stably express different fluorescent reporter proteins

**Fig 1:**
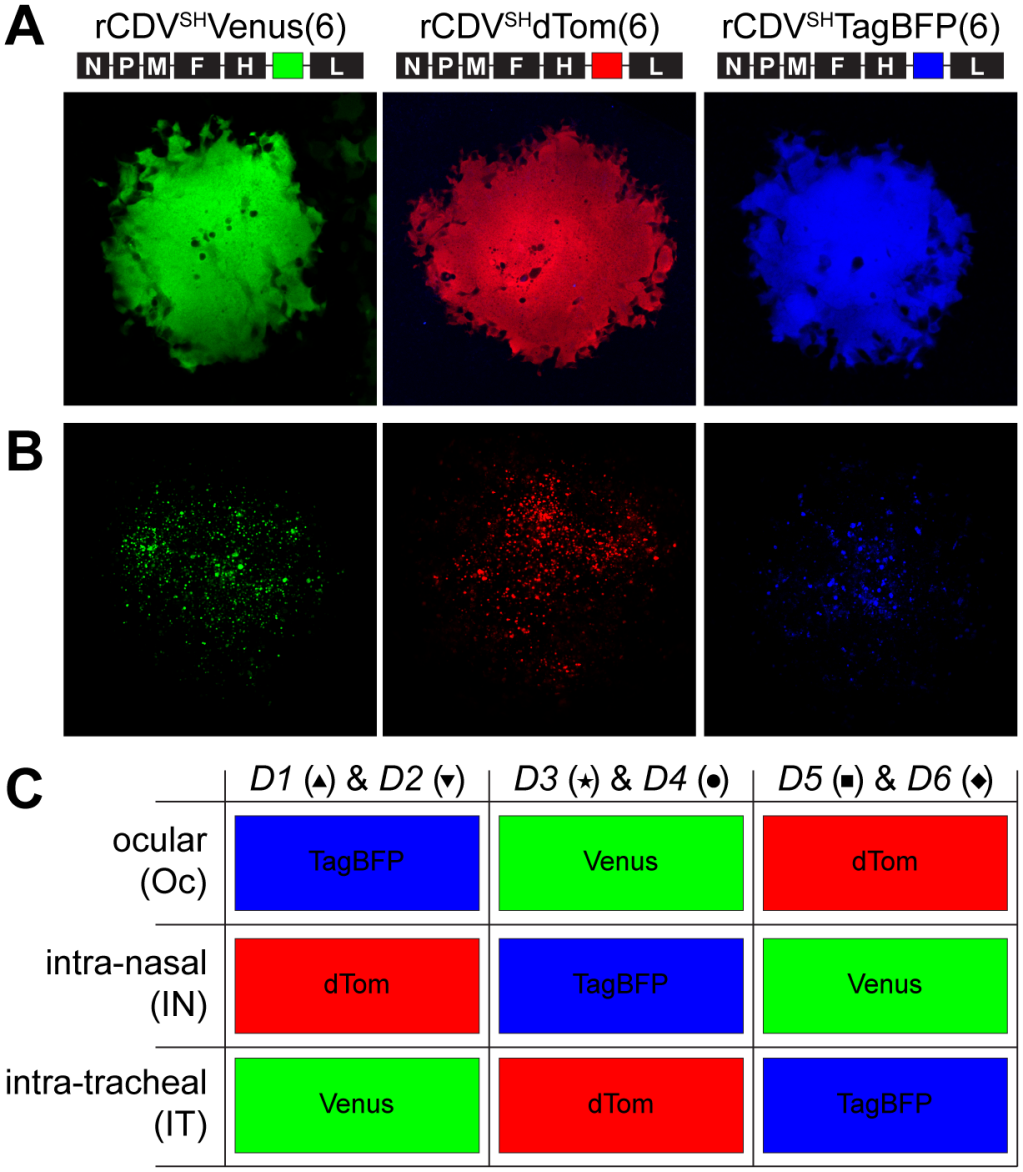
*In vitro* validation and *in vivo* experimental design. (A, top panel) Three rCDVs expressing a fluorescent reporter protein as an ATU from the 6^th^ position in the genome were generated (rCDV^SH^Venus(6), rCDV^SH^dTom(6), rCDV^SH^TagBFP(6)), rescued and (A, bottom panel) grown on Vero-cCD150 cells. (B) rCDVs were shown to efficiently infect ConA-stimulated ferret WBC. (C) Donor ferrets 1–6 (*D1-6*) were simultaneously inoculated with a low dose of the three indicated rCDVs delivered via Oc, IN and IT inoculation in different administration route combinations, divided over three sequential experiments.

Plasmids containing full-length CDV strain Snyder-Hill (CDV^SH^) antigenomes were modified to encode rCDVs expressing fluorescent reporter proteins from an additional transcription unit (ATU). Three fluorescent reporter proteins (Venus, dTom and TagBFP) were selected on basis of spectral discernibility, brightness, photo-stability, lack of oligomerization and potential to be detected by in-house flow cytometer and confocal microscope. These viruses, rCDV^SH^Venus(6), rCDV^SH^dTom(6) and rCDV^SH^TagBFP(6) were rescued and grown on Vero cells modified to express canine (c) CD150 (Vero-cCD150) in which they produced high levels of fluorescence in multinucleated syncytia (Fig 1A). Infection of concanavilin A (ConA)-stimulated white blood cells (WBC) isolated from CDV-naive ferrets resulted in large numbers of fluorescent cells, as detected by confocal laser scanning microscopy (CLSM, Fig 1B) and flow cytometry.

### Simultaneous inoculation with three rCDV^SH^s causes lethal disease in ferrets

**Fig 2:**
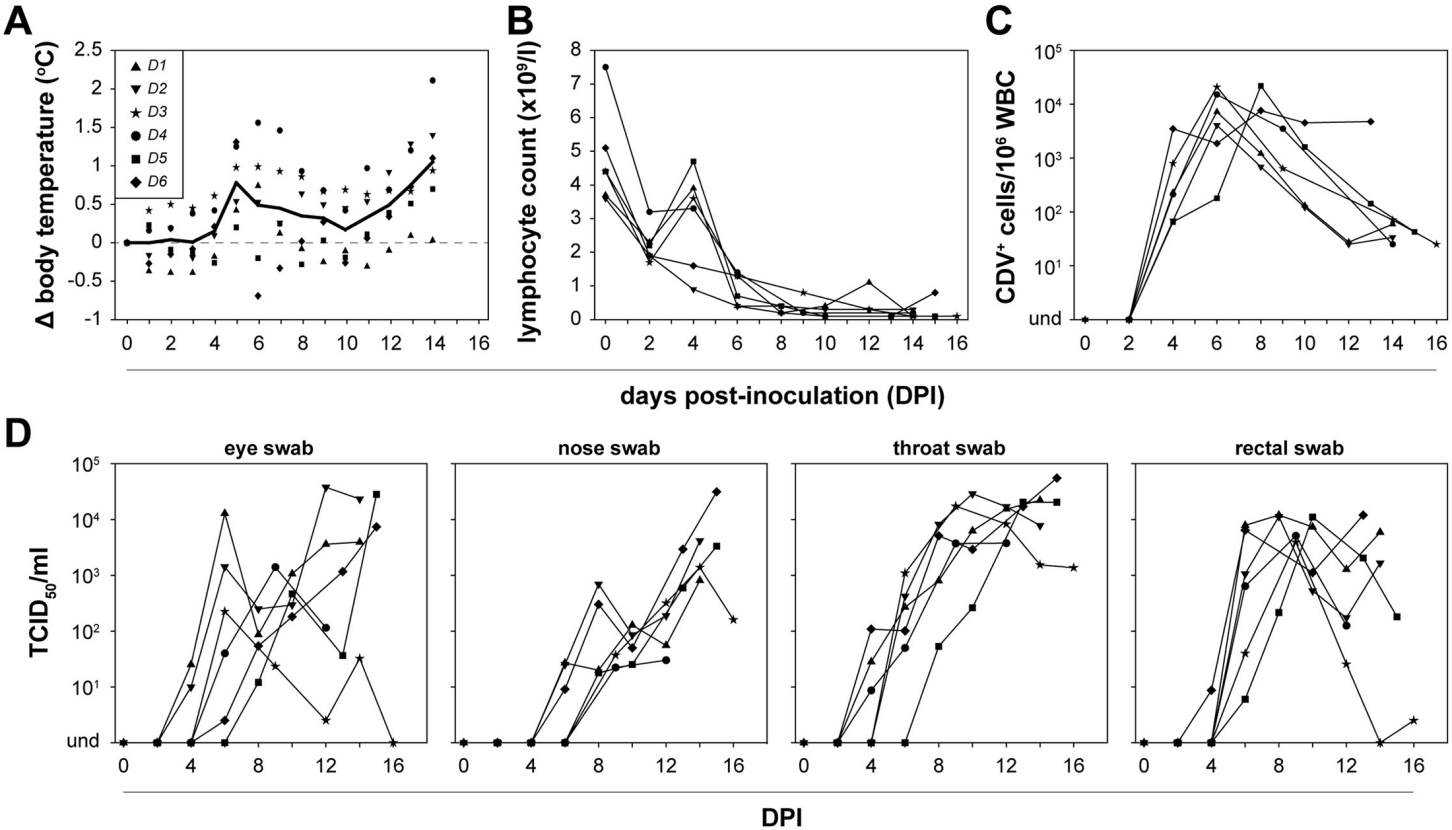
Inoculation with rCDVs leads to systemic infection and disease. Simultaneous inoculation of donor ferrets (*D1-6*) via multiple administration routes with three reporter viruses resulted in systemic disease. (A) Body temperature was measured using an intra-peritoneal probe and a biphasic fever pattern was observed. (B) WBC counts measured in EDTA blood showed that animals became lymphopenic. (C) Systemic virus replication was detected by virus isolation from WBC on Vero-cCD150 cells, while (D) local virus replication was demonstrated as virus isolation from eye, nose, throat and rectal swabs.

Six donor ferrets (*D1* –*D6*) were simultaneously inoculated in three consecutive experiments via the ocular (Oc), intra-nasal (IN) and intra-tracheal (IT) routes with a low dose of the indicated rCDV^SH^ (3.3 x 10^3^ TCID_50_/route), depositing viruses in the eyes, nose or lungs. Even though the viruses had been engineered to have identical genome lengths to prevent subtle differences in fitness impacting the outcome of the *in vivo* experiment, the rCDVs were alternated over the three route combinations to mitigate any unforeseen effect on pathogenesis (Fig 1C). All donor ferrets developed fever with a biphasic pattern, with temperature peaks around 5–7 and 12–14 days post-inoculation (DPI) (Fig 2A). The first fever peak corresponded with the onset of lymphopenia: peripheral lymphocyte counts were strongly reduced by the end of the first week after infection, and did not recover during the second week (Fig 2B). All donor ferrets became lethargic and were euthanized 14–16 DPI. Virus could be isolated from WBC of all ferrets from 4 DPI, with peak viremia levels observed around 6–8 DPI (Fig 2C). The decline in viral load in WBC during the second week was likely due to the virtual absence of lymphocytes in peripheral blood, resulting in exhaustion of susceptible CD150-positive cells. Virus was isolated from eye, nose, throat, and rectal swabs, in most ferrets with progressively increasing virus loads during the second week after inoculation (Fig 2D).

### Simultaneous inoculation of ferrets with three rCDVs results in systemic replication of one or multiple viruses

**Fig 3:**
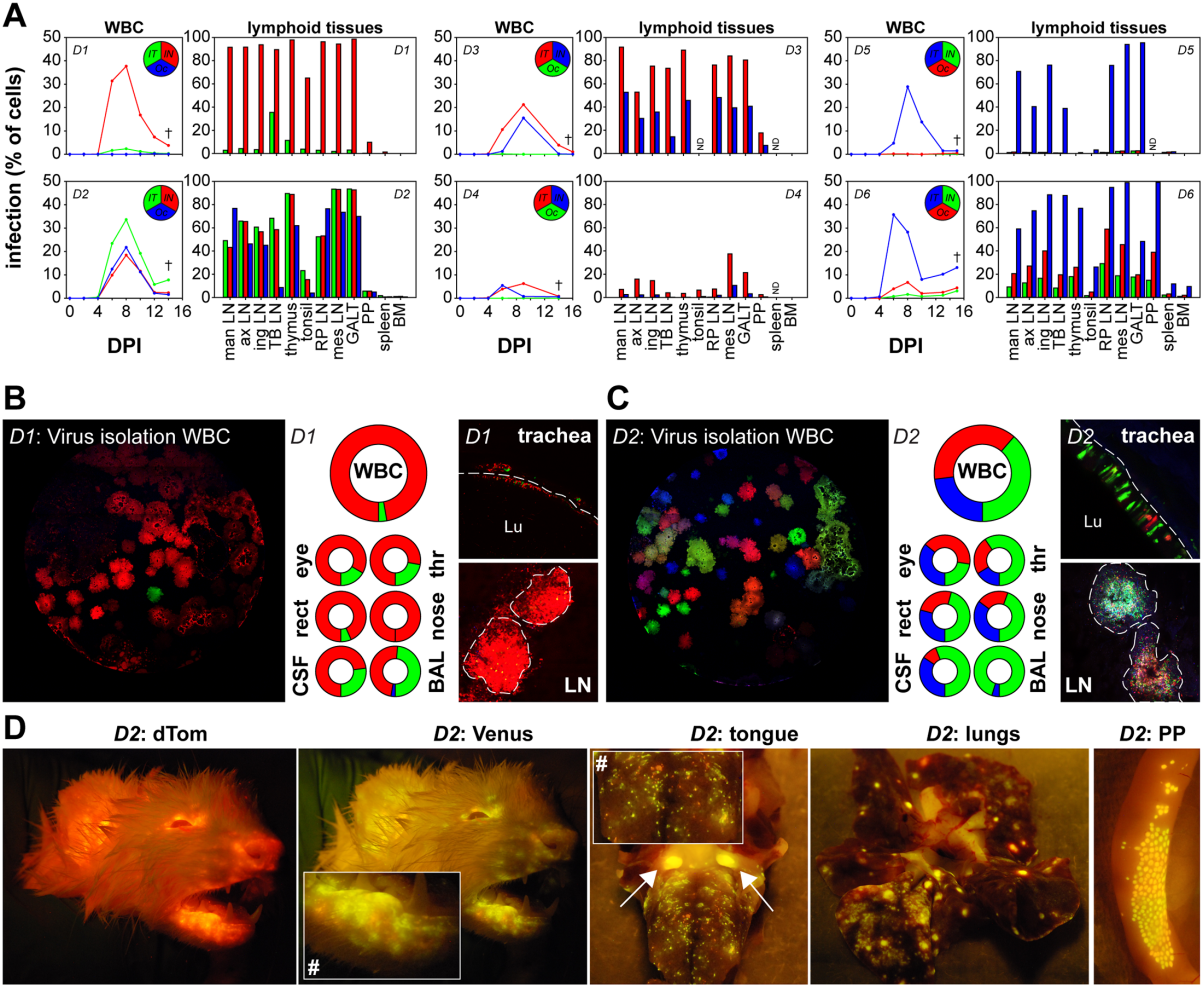
Microscopic and macroscopic detection of fluorescence in donor ferrets. (A) WBC infection percentages of the rCDVs in directly inoculated (*D1-6*) ferrets were determined by flow cytometry at different time-points. The circular inset shows which reporter virus was administered to a donor ferret via which route. After necropsy, infection percentages of the rCDVs in single cell suspensions of lymphoid tissues were determined by flow cytometry. rCDVs were detected in all lymphoid tissues (man LN: mandibular lymph node; ax LN: axillary LN; ing LN: inguinal LN; TB LN: tracheo-bronchial LN; RP LN: retropharyngeal LN; mes LN: mesenteric LN; GALT: gut-associated lymphoid tissue; PP: Peyer’s patches; BM: bone marrow; ND: not determined). Results obtained from lymph nodes corresponded to the kinetics of the rCDVs in WBC. In 5/6 ferrets the IT delivered virus became dominant (*D2 –D6*). (B, C) Donor ferrets *D1* and *D2* were chosen as representative examples. Virus was isolated from WBC, BAL, CSF and throat, nose, eye and rectal swabs by titration on Vero-cCD150 cells. (Left panels) Screening of virus isolations by CLSM allowed determination of the dominance of rCDVs at 8 DPI. (Centre panels) Relative contribution of the various reporter viruses to the total amount of rCDV present in the sample collected 8 DPI or at necropsy (CSF and BAL). (Right panels) Direct CLSM of various tissues at necropsy revealed fluorescence in the epithelium of the trachea (dotted line marks basement membrane, Lu marks lumen), and B-cell follicles in lymphoid tissues (delineated by dotted line). (D) Macroscopic fluorescence produced by rCDV^SH^Venus(6) and rCDV^SH^dTom(6) was directly detected in live ferrets and during necropsy at the nose, eyes, mouth and skin. The inset in the second panel clearly shows that green and red foci of infected cells can be discerned (#). During necropsy, macroscopic fluorescence was detected in the tongue (arrows show tonsils), lungs and Peyer’s patches. Again, the inset in the third panel shows separate foci of green and red cells (#).

Expression of fluorescent proteins within WBC throughout the course of the experiment, or in single cell suspensions prepared from lymphoid tissues collected at necropsy, was assessed by flow cytometry (Fig 3A). In one ferret (*D5*) only one fluorescent protein was detected, in three ferrets (*D1*, *D3*, *D4*) a combination of two fluorescent proteins was detected and in two ferrets (*D2*, *D6*) all three inoculated viruses had initiated productive infections as shown by simultaneous detection of red, green and blue fluorescent proteins. Interestingly, in ferrets showing systemic infection with two or three viruses, all fluorescent proteins were already detectable in WBC collected at 6 DPI. This demonstrates that even though viruses were administered via multiple inoculation routes, parallel onset of viremia occurred within one week after inoculation.

### rCDV uses multiple routes of entry in parallel

In all donor ferrets IT inoculation resulted in viremia, confirming that direct inoculation into the lower respiratory tract is a highly efficient entry route for morbilliviruses (Fig 3A). In five out of six donor ferrets, IN inoculation also resulted in viremia, demonstrating that morbilliviruses can also efficiently invade a host when 3300 TCID_50_ in a low volume (25μl/nostril) was deposited in the upper respiratory tract (Fig 3A). Finally, in two out of six ferrets the morbillivirus directly inoculated into the conjunctival sac behind the lower eyelid (again using a low volume inoculum) caused viremia, demonstrating this is a functional route for morbillivirus entry, although apparently less effective than entry through the respiratory route (Fig 3A).

### Multi-route delivery of rCDVs results in widespread systemic replication

Next we focused on the period between onset of viremia and euthanasia of the donor ferrets to assess morbillivirus dissemination. In one ferret (*D1*) the virus inoculated via the IN route became dominant, while in all other ferrets the virus given by IT inoculation became dominant. Likewise, viruses that predominated in WBC were in most cases also present at the greatest level in single cell suspensions from lymphoid tissues (Fig 3A).

Detection of fluorescent protein expression in WBC (Fig 3A) showed a good correlation with virus isolation from WBC (Fig 3B and 3C, left panel). Ferrets *D1* and *D2* illustrate this since these animals represented two extremes of the study. In ferret *D1* two viruses were detected systemically (rCDV^SH^Venus(6) and rCDV^SH^dTom(6)), however one virus (rCDV^SH^dTom(6)) became dominant. In ferret *D2* all three viruses replicated at comparable levels. Virus isolation from WBC in Vero-cCD150 cells resulted in predominantly red plaques for *D1*, and in multicolored plaques for *D2*. Photomicrographs in the left panels of Fig 3B and 3C show a high resolution CLSM image of a representative well, while the large pie charts show the relative color distribution produced by viruses isolated from WBC collected at 8 DPI. The smaller pie charts show the color distribution of virus isolated from other samples, including swabs of the conjunctivae, nose, throat and rectum collected at 8 DPI (see also Fig 2D), and cerebrospinal fluid (CSF) and broncho-alveolar lavage (BAL) collected at necropsy. Interestingly, the proportion of green fluorescent virus in ferrets *D1* and *D2* was higher in BAL than in all other tissues, corresponding to IT inoculation of these two ferrets with rCDV^SH^Venus(6). In ferret *D1*, low systemic detection of green fluorescent cells, was contrasted by a relatively high percentage of green fluorescent cells in the tracheo-bronchial lymph node (TB LN) which drains the lungs (Fig 3A). At necropsy, CLSM analysis of respiratory tract tissues and lymph nodes of ferret *D1* revealed a predominance of red fluorescent cells (Fig 3B, right panels), whereas the same tissues collected from ferret *D2* contained a mixture of different colored fluorescent cells (Fig 3C, right panels).

Macroscopic fluorescence produced by rCDV^SH^Venus(6) and rCDV^SH^dTom(6) was imaged both in living ferrets and at necropsy. Macroscopic detection of blue fluorescence was not technically possible using the available apparatus. Both green and red fluorescence were detected in the head of ferret *D2* (Fig 3D, fluorescence was detected mainly at the nose, eyes, mouth and skin). Use of the light emitting diode (LED) lamp to detect green fluorescence, allowed detection of separate green and red foci of infected cells (Fig 3D, inset in second panel at (#)). Macroscopic imaging of tissues during necropsies is illustrated by photos of the tongue, lungs and Peyer’s patches (PP) of ferret *D2*, again showing a clear separation of green and red fluorescent foci of infection (Fig 3D, inset in third panel at (#)).

### Double- and triple-rCDV-infected cells *in vivo* during dissemination

**Fig 4:**
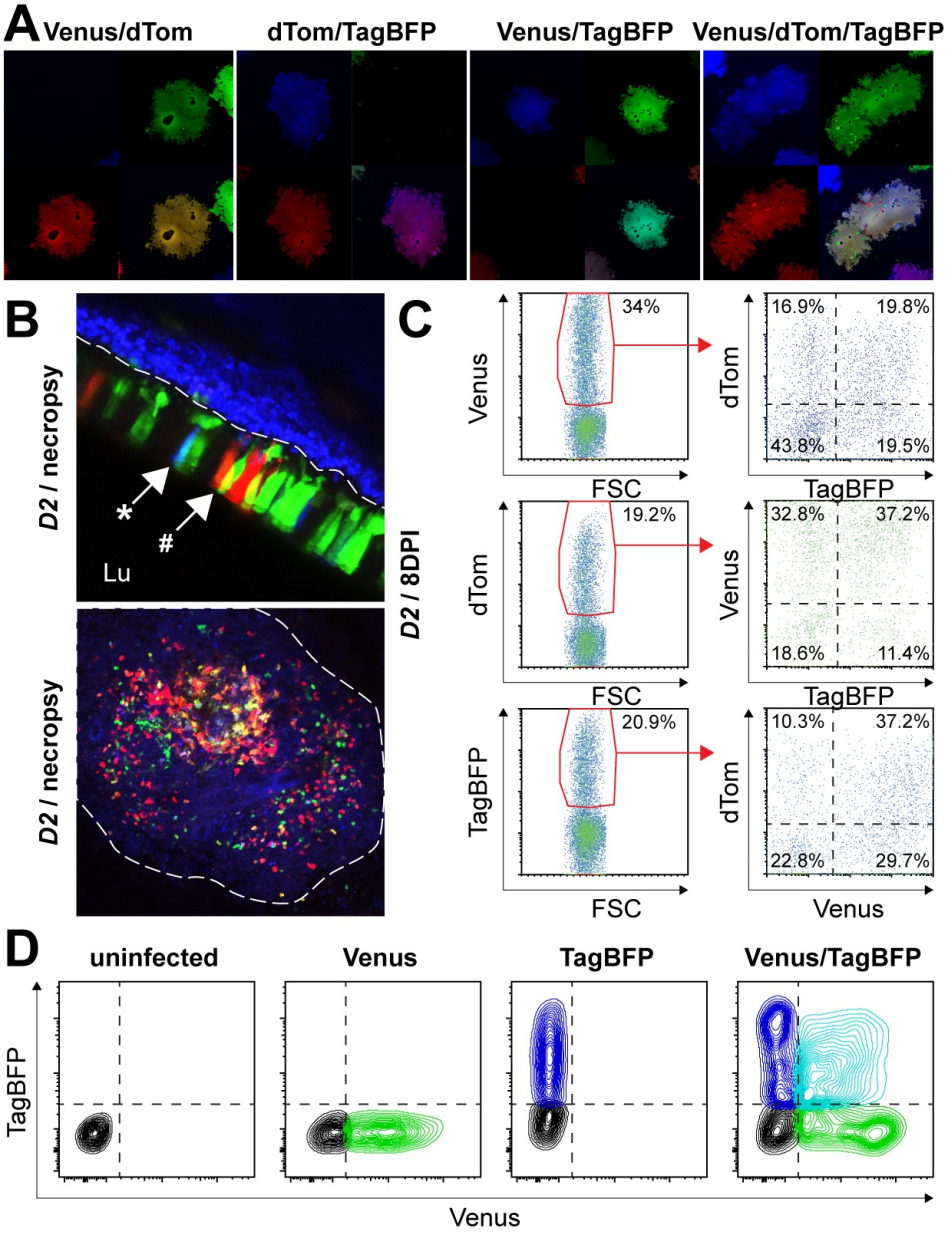
Detection of rCDV double- and triple-infected cells. (A) Virus isolation from WBC by titration on Vero-cCD150 cells revealed syncytia that were simultaneously positive for two reporter proteins (*i*.*e*. Venus/dTom [yellow], dTom/TagBFP [purple] or Venus/TagBFP [cyan]) or all three reporter proteins (Venus/dTom/TagBFP [white]). (B, upper panel) Direct confocal analysis of the trachea from ferret *D2* showed that single infected cells (Venus, dTom or TagBFP) were dominant, but sparse Venus/TagBFP and Venus/dTom double-positive cells were detected as cyan (*) or yellow (#), respectively. (B, lower panel) Direct confocal analysis of the spleen from ferret *D2* revealed the presence of single-infected and all possible combinations of double-infected cells. (C) Direct flow cytometry of WBC collected from ferret *D2* on 8 DPI indicated that single-, double- and triple-positive cells were present in peripheral blood. Similar results were obtained at different time-points. (C, upper panel) When Venus^+^ cells were selected (as indicated by red gate), dTom^-^/TagBFP^-^ (single-infected cells), dTom^+^/TagBFP^-^ or dTom^-^/TagBFP^+^ (double-infected cells) and dTom^+^/TagBFP^+^ (triple-infected) cells were detected. (C, middle and lower panel) Similar results were obtained when the analysis was performed by initially gating dTom^+^ or TagBFP^+^ cells. Percentages of positive cells are indicated in the plots. (D) Canine lymphoma B-cells (CLBL-1) were inoculated with rCDV^SH^Venus(6) and/or rCDV^SH^TagBFP(6) in the presence of infection-enhancing lipopeptide Pam3CSK4. Green and blue fluorescence levels were determined 24 hours post infection by flow cytometry. Venus and TagBFP single positive cells are shown in green and blue, respectively, while double-positive cells are shown in cyan.

Morbillivirus viremia is mediated by circulation of infected cells rather than cell-free virions. As a consequence, morbilliviruses predominantly disseminate by cell-to-cell spread within the host. It has been hypothesized that superinfection immunity is a significant barrier to dual infections of cells. Unexpectedly, during virus isolation procedures from WBC we not only observed green, blue or red syncytia, but also syncytia that contained two or three fluorescent proteins, pseudo-colored as yellow (Venus / dTom), purple (dTom / TagBFP), cyan (Venus / TagBFP) or white (Venus / dTom / TagBFP) following image acquisition by CLSM (Fig 4A). Since it may be possible that double- and triple-positive syncytia could have been caused by fusion of single-infected cells, thus not representing true double or triple infections of single cells, we performed direct CLSM of tissues collected during necropsy. This confirmed the presence of double-infected cells in the epithelium of the trachea (Fig 4B, upper panel, Venus / TagBFP cell shown as cyan [*] and Venus / dTom cell shown as yellow [#]) and in the spleen (Fig 4B, lower panel). In the spleen, single-infected cells were observed throughout the section in red, green and blue, whereas many double-positive cells were observed in cyan, yellow and purple. Finally, double and triple infections were confirmed by flow cytometry. As an example, WBC obtained from ferret *D2* at 8 DPI were gated for positive events for a single reporter protein (Fig 4C, left panels), for which subsequently the other two reporter proteins were plotted (Fig 4C, right panels). This demonstrated the presence of single-infected cells (lower-left quadrant), but double- (lower-right and upper-left quadrant) and triple-infected (upper-right quadrant) lymphocytes were also commonly detected. Although triple-infected cells were only detected in 2/6 animals, double-infected cells were more common and were detected in 5/6 animals. To confirm these *in vivo* observations, we performed *in vitro* competition experiments in canine B-lymphoblastoma (CLBL-1) cells [41], and confirmed that double infections were also achieved *in vitro* (Fig 4D). Moreover, *in vitro* double infections were also observed when the second virus infection was performed six hours after the first infection (S1 Fig).

### rCDV dissemination results in widespread infection of the nasal cavity and CNS

**Fig 5:**
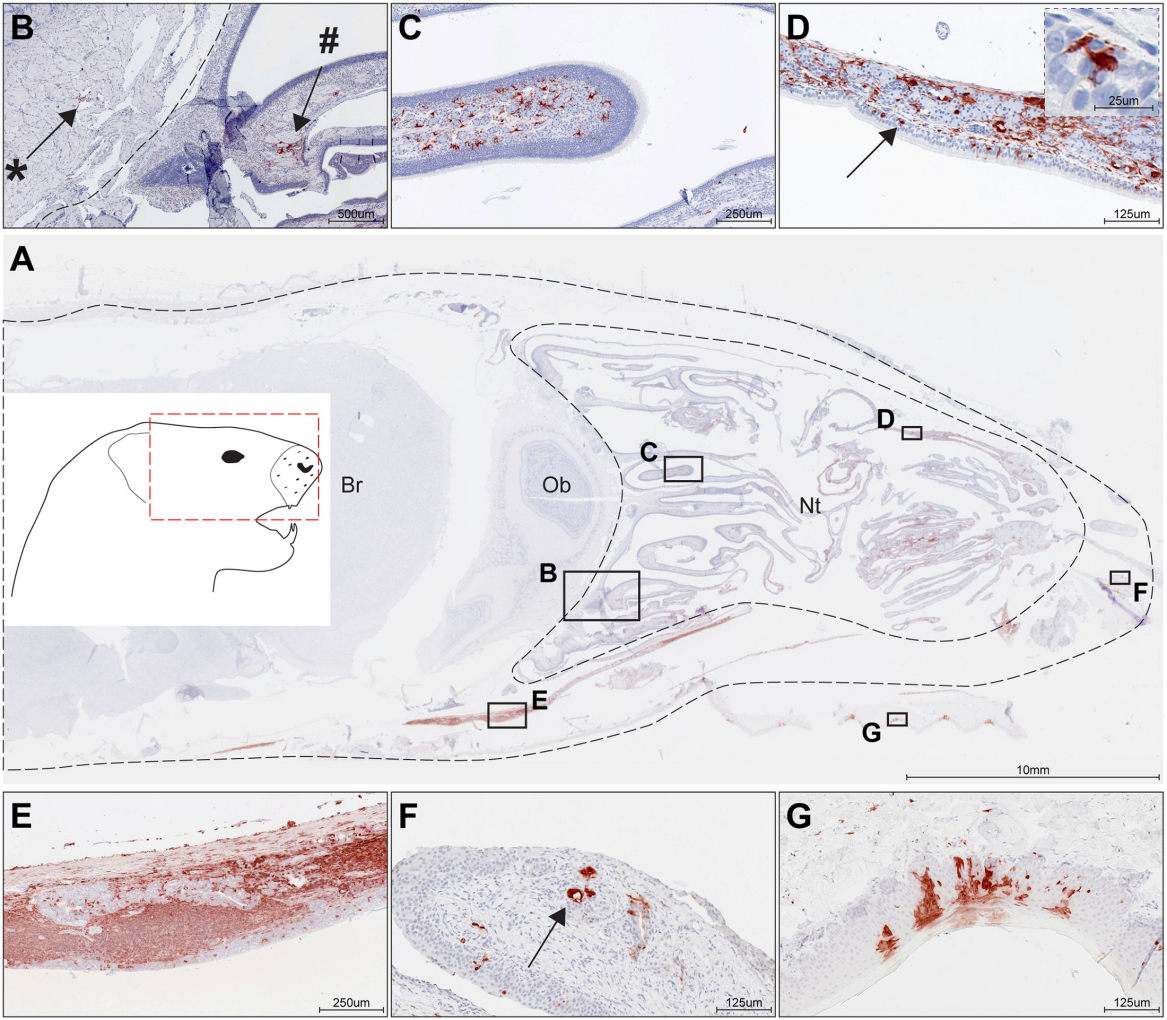
rCDV was detected in the nasal cavity of directly inoculated ferret *D4*. Immunohistochemistry detecting CDV was performed on (A) complete ferret head sections (red box in inset shows anatomic location of section) and showed abundant rCDV in the nasal turbinates (Nt, delineated by dotted line). In this ferret rCDV was only observed in few meningeal cells and not in the cerebrum (Br) and olfactory bulb (Ob). In general, CDV-positive cells were mainly found in the submucosa of the respiratory and olfactory mucosa and nasal-associated lymphoid tissues (NALT). (B) Cells surrounding the nerve twigs of the olfactory nerve were CDV-positive on both sides of the cribriform plate (dotted line), on both the side of the nasal cavity (#) and the olfactory bulb (*). (C) Few CDV-positive cells were detected in the olfactory epithelium, and many CDV-positive cells were present in the submucosa of the olfactory epithelium. Positive cells had an irregular macrophage-like morphology. (D) CDV-positive cells were detected in the respiratory epithelium of the nasal turbinates, although most positive cells within the epithelium appear non-epithelial (arrows and insert). Again, CDV-positive cells were abundant in the submucosa and had an irregular macrophage-like morphology. (E) Positive stretch of CDV-positive cells, including NALT with infected lymphocytes and fibroblasts. CDV-positive macrophages were also detected in the epithelial layer. (F) Submucosal nasal glands stained occasionally positive for CDV predominantly in the tip of the nose (arrow). (G) Squamous epithelium of the soft palate stained positive for rCDV.

Since airborne transmission of respiratory viruses is assumed to be associated with virus shedding from the upper respiratory tract [42, 43], we assessed the distribution of CDV-infected cells in the nasal cavity of ferrets *D4* (Fig 5) and *D5* (S2 Fig). Since CDV^SH^ is highly neurotropic, we performed immunohistochemical analysis of slides containing a sagittal section of the entire ferret head which permitted assessment of infected cells in both the nasal cavity and brain. CDV-infected cells were mainly detected in the nasal cavity of ferret *D4* (Fig 5A), and few positive cells were observed in the meninges. Cells surrounding the nerve twigs of the olfactory nerve were CDV-positive on both sides of the cribriform plate (dotted line), on both the side of the nasal cavity (#) and the olfactory bulb (*) (Fig 5B). Very few cells within the olfactory epithelium were CDV positive (Fig 5C), whereas the respiratory epithelium contained many CDV-infected cells (Fig 5D), including ciliated epithelial cells and macrophage-like cells (Fig 5D, macrophages indicated by arrow and shown in inset). Throughout the nasal cavity the majority of CDV-positive cells was found in the submucosa and included fibroblasts, large macrophage-like cells, and lymphocyte-like cells. In the nasal-associated lymphoid tissues (NALT) the majority of lymphoid cells were CDV positive (Fig 5E). In addition, CDV- infected glands were occasionally observed in the nasal cavity, mainly in the tip of the nose (Fig 5F). CDV-infected squamous epithelial cells were observed in the soft palate (Fig 5G), mimicking similar observations in MV-infected macaques.

In ferret *D5*, rCDV was detected in the nasal cavity and CNS (S2A Fig). In the nasal cavity, the number of CDV positive cells was lower in comparison to ferret *D4*, and consisted predominantly of fibroblasts and macrophage-like cells in the submucosa. In the CNS, CDV-infected cells were frequently observed in the meninges, predominantly in the meninges surrounding the cerebellum. Positive ependymal cells were detected in the choroid plexus, responsible for the production of CSF (S2B Fig), which corresponds to the isolation of virus from the CSF from this ferret. In the cerebellum, meninges surrounding the cerebellum and brainstem, endothelial cells and cells adjacent to blood vessels were occasionally CDV positive (S2C Fig). This suggests hematogenous spread of CDV into the CNS. In both the olfactory bulb and cerebrum foci of CDV positive cells, including neurons and glial cells, were detected (S2D and S2E Fig). Finally, CDV-positive cells were observed within the bone marrow.

### rCDV infection of donor ferrets results in airborne transmission to recipient ferrets

**Fig 6:**
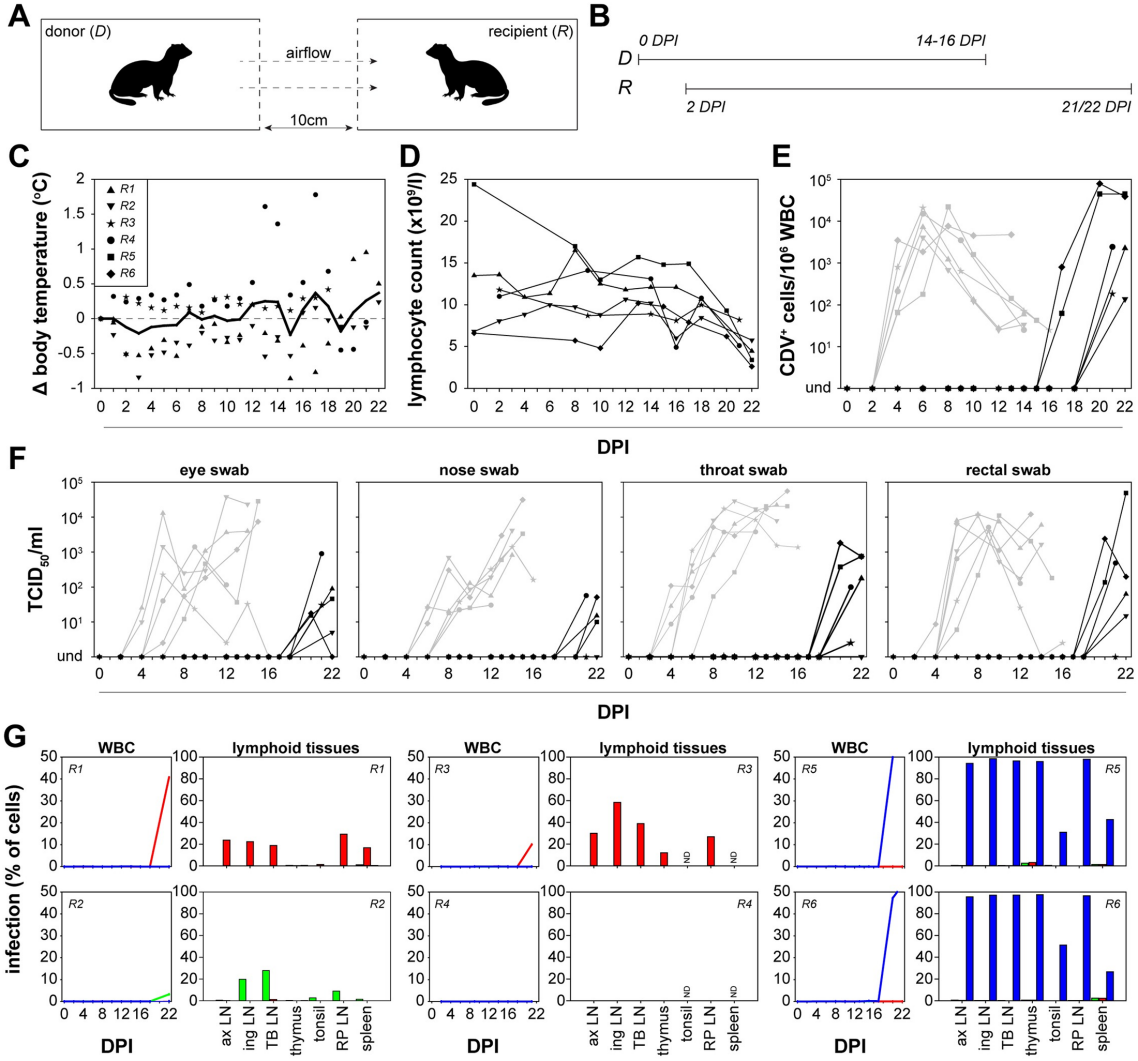
*In vivo* transmission of rCDV to recipient ferrets. (A, B) Susceptible recipient ferrets (*R1-6*) were placed pairwise in neighboring cages of the donor ferret at 2 DPI. Arrows indicate airflow. All directly inoculated ferrets were euthanized at 14 to 16 DPI, whereas recipient ferrets were euthanized 21 or 22 DPI. (C) Fever was rarely detected in recipient ferrets, but these animals developed (D) lymphopenia and (E) systemic virus replication as detected by virus isolation from WBC as well as (F) local virus replication demonstrated as virus isolation from eye, nose, throat and rectal swabs. In panels E and F, grey lines represent the respective virus loads in donor ferrets (as shown in Fig 2). Transmission was detected in 6/6 animal pairs. (G) WBC infection percentages of the rCDVs in recipient ferrets were determined by flow cytometry at different time-points. After necropsy, infection percentages of the rCDVs in single cell suspensions from lymphoid tissues were determined. rCDV was detected in most lymphoid tissues (ax LN: axillary LN; ing LN: inguinal LN; TB LN: tracheo-bronchial LN; RP LN: retropharyngeal LN; ND: not determined). Results obtained from lymph nodes corresponded to the kinetics of the rCDVs in WBC. In 6/6 ferrets the single-color virus dominant in the donor animal transmitted to the recipient ferret.

To assess airborne transmission, CDV-naive recipient (*R1 –R6*) ferrets were placed in transmission cages at 2 DPI. Donor and recipient ferrets were sampled every other DPI and followed for a maximum of 16 or 22 DPI respectively (Fig 6A and 6B). Ferrets were housed in pairs, meaning that recipient ferret *R1* was placed in a cage adjacent to donor ferret *D1*. Body temperature was measured in 4 out of 6 recipient ferrets. Fever was not observed in the recipient ferrets tested up to the time point of euthanasia (Fig 6C). However, the majority of recipient ferrets showed decreased lymphocyte counts shortly before euthanasia (Fig 6D). Airborne transmission was confirmed in all donor-recipient pairs and infectious rCDV was isolated from WBC (Fig 6E), nose, throat, eye and/or rectal swabs collected from recipient ferrets (Fig 6F). By using flow cytometry, rCDV replication was detected in WBC of 5 out of 6 ferrets (Fig 6G). For recipient ferret (*R4*) in which CDV-infected cells were not detected by flow cytometry, rCDV^SH^dTom(6) was isolated from throat and eye swabs. In all cases, only a single colored rCDV transmitted to the recipient ferret, which was also found in the lymphoid tissues at euthanasia and always was the virus that predominated in the corresponding donor animal (Fig 3A).

## Discussion

We have performed *in vivo* competition and transmission studies in ferrets with virologically identical but spectrally distinct rCDVs administered simultaneously via multiple routes. Our aim was to study the temporal and spatial interplay of viruses during the early, intermediate and late stages of CDV infection. These “rainbow CDV” studies are the first to show that morbilliviruses can use multiple entry routes in parallel. Detection of circulating or lymphoid tissue-derived lymphocytes expressing multiple fluorescent reporter proteins was common, demonstrating that *in vivo* superinfection immunity is not a restrictive phenomenon. Airborne transmission to recipient ferrets was detected in all animal pairs, underpinning the highly infectious nature of morbilliviruses.

Animal models of human viral diseases provide the bedrock of much of the current understanding of tropism and pathogenesis, and are essential for the development and licensure of drugs and vaccines. Ideally the human pathogen under study should not need to be adapted to the animal and the disease process should recapitulate the full clinical spectrum in people. This is challenging and oftentimes either the pathogen or host must be genetically altered to produce disease. Therefore, related animal pathogens infecting natural host species provide useful surrogates in the pathogenesis toolkit. For example, Sendai virus infection of mice [44], bovine respiratory syncytial virus infection of calves [45] and simian immunodeficiency virus infection of NHPs [46] have provided important insights into the pathogenesis of closely related human viruses. Likewise, experimental CDV infections of ferrets have provided important insights into the pathogenesis of MV and other morbilliviruses [47–50].

Both measles [51] and canine distemper [47] are recognized as highly infectious diseases that are spread via the respiratory tract. The availability of rMVs expressing fluorescent proteins allowed us to identify alveolar macrophages and DCs in NHPs as early target cells following aerosol inhalation [24]. In that study, substantial numbers of MV-infected cells were not detected in the upper respiratory tract even though minuscule numbers of macrophages and DCs were detected in the deep lung at early time points after inoculation. This fitted well with our experience of IT inoculation as a highly reliable and standardized route of experimental MV infection [26]. The majority of CDV pathogenesis studies in ferrets has used IN inoculation, delivering the virus to the upper respiratory tract. Although it is important to note that IN inoculation of relatively large volumes can easily result in deposition of virus in both the upper and lower respiratory tracts [52], we also hypothesized that it was unlikely that morbilliviruses exclusively used the lower respiratory tract as a portal of entry [30]. Therefore, we simultaneously inoculated ferrets with multicolor rCDVs via the IN and IT routes, ensuring delivery to only the upper and lower respiratory tract, respectively. Since the conjunctivae have also been suggested as a portal of entry for many respiratory viruses [33], including MV [32], ocular inoculation was used as a third possible route of entry. IN and IT delivery resulted in viremia in 5 out of 6 and 6 out of 6 ferrets, respectively, within a week after inoculation. The fact that the IT-delivered virus became dominant in 5 out of 6 ferrets suggests that this is the most efficient port of entry, confirming previous observations with MV infection of NHPs [26]. This could be explained by the fact that in the lungs the potential target cells (alveolar macrophages and/or DCs) are directly accessible and not shielded by an epithelial barrier. However, since virus delivered by IN inoculation in a low volume also resulted in viremia in the majority of the ferrets, this demonstrated that multiple routes of entry can be used in parallel. Inoculation of the virus onto the conjunctivae resulted in infection in only 2 out of 6 ferrets, demonstrating that this is a legitimate, albeit less efficient entry portal. However, since there is a direct connection between the eyes and the upper respiratory tract via the nasolacrimal duct, transport of virus particles to the upper respiratory tract after conjunctival delivery cannot be excluded. Moreover, it is important to note that this study was not designed to assess statistically significant differences between these routes of entry.

CDV dissemination is mediated by infected circulating and tissue-resident B- and T-lymphocytes [35], and results in lymphopenia and fever. During dissemination the infection remains highly cell-associated, and few virions are detected in plasma. Spread is mediated mostly by direct cell-to-cell transmission, e.g. by the formation of virological synapses [34, 53, 54]. Here, we show that regardless of the route of entry, all donor ferrets developed a similar course of fatal disease, and severity was independent of the number of circulating rCDVs. Interestingly, significant numbers of lymphocytes co-expressed two or three fluorescent reporter proteins, demonstrating that they were infected with viruses inoculated at different sites. Although it has been shown that morbillivirus infection induces superinfection immunity *in vitro* [38], the incredibly rapid dissemination of CDV (with percentages of infected WBC rising from undetectable to more than 50% within 2 days in some animals) leads to lymphocytes being double- or triple-infected. We consider serial, direct cell-to-cell transmission of rCDVs from different donor cells to single acceptor cells the most likely explanation of the existence of double or triple infections. In fact, double infection was readily reproduced *in vitro* by exposing a B-lymphoblastic canine cell line to two rCDVs, and was also reproduced when the second infection was performed six hours after the first.

Multi-route delivery of virologically identical but phenotypically distinct viruses is a powerful approach to dissect the complex interplay in evolving pathogenesis. None of the data suggest that the expression of different fluorescent reporter proteins had an effect on virus fitness and we have done our utmost to ensure that rCDVs were genetically identical in terms of genome length and ATU design. However, we cannot exclude the possibility that genes encoding Venus, dTom and TagBFP contain secondary or tertiary RNA structures or immune-activating sequences. Alternating the rCDVs and administration routes in three subsequent experiments further mitigated this risk.

Although epidemiological observations have demonstrated that CDV is a highly contagious virus, surprisingly few experimental studies have examined transmission. In 1926, Dunkin and Laidlaw meticulously described precautions taken to perform experimental CDV infections of ferrets and keep their breeding stock of animals free of canine distemper [47]. Three transmission pathways: direct contact between sick and healthy animals, housing a healthy animal in a cage from which a moribund animal had been removed several hours earlier, and airborne transmission were described [47]. The timespan between onset of disease in the donor and recipient animals was around ten days. Subsequent direct transmission studies documented a period of 6 to 11 days for development of disease in recipient animals [55] and experimental CDV outbreak studies in ferrets support a role for airborne transmission [56]. The observed time period required for CDV transmission in our experiment was in good accordance with these previous reports [47, 56].

Even though we could not determine the exact tissue origin of transmitted virus due to widespread dissemination of different colored viruses throughout the donor ferrets, the dominant virus of the donor always was the only virus transmitted to the recipient. The added value of our study was that the transmission of a single color rCDV, even when two or three rCDVs were detected in donor ferrets, suggested a bottleneck event during airborne transmission: apparently only one or a few infectious units were transmitted from donor to recipient ferret. This mirrors influenza airborne transmission between ferrets, in which virus populations in recipient ferrets proved to be genetically much more homogeneous than those of the donor ferrets [43, 57, 58]. However, in this model transmission was restricted to airborne transmission, so the situation could be different for indirect and direct contact transmission. Overall, this illustrates the utility of gaining a comprehensive understanding of transmission in this important animal model, which is also being developed for Ebola virus and Nipah virus [59–61].

Here, we have performed *in vivo* competition and transmission studies with rCDVs discernable on basis of their fluorescent reporter proteins. We believe that this model will prove its use in the future for *in vivo* competition studies to identify factors associated with viral fitness.

## Materials & methods

### Ethics statement

Animal experiments were conducted at Erasmus MC, in strict compliance with European guidelines (EU directive on animal testing 2010/63/EU) and Dutch legislation. The study protocol was approved by Stichting Dier Experimenten Commissie Consult (DEC Consult, permit number EMC3043), a Dutch independent animal experimentation ethics review board. The manuscript was prepared in accordance with the ARRIVE guidelines [62].

CDV-naive ferrets were housed in groups prior to rCDV infection, received standard feed on a daily basis and had access to water *ad libitum*. Cages contained several sources of environmental enrichment. During the infection and transmission studies, ferrets were housed in transmission cages [63]. Briefly, ferrets were housed individually in perspex cages, with paired donor (*D*) and recipient (*R*) ferrets being separated by two stainless steel grids. The bottom of the cages was covered with carpet and cages were not cleaned during the course of the experiment to prevent aerosolizing bodily excretions. Transmission cages were placed in HEPA-filtered, negatively pressurized biosafety level 3 (BSL-3) isolators. Animal welfare was checked on daily basis, and all animal handling was performed under light anesthesia using ketamine and medetomidine. After handling, atipamezole was administered to antagonize the effect of medetomidine.

### Cells

Vero cells stably expressing the CDV receptor canine SLAM (Vero-cCD150) (kind gift of Dr. Y. Yanagi, Kyushu University, Fukuoka, Japan) were cultured as described previously [64]. To obtain primary ferret white blood cells (WBC), small-volume blood samples were collected from CDV-naive ferrets in Vacuette tubes (Greiner) containing K_3_EDTA as an anticoagulant. Red blood cells in blood were subsequently lysed with red blood cell lysis buffer (Roche, Basel, Switzerland), washed and resuspended in complete RPMI 1640 medium (Gibco Invitrogen, Carlsbad, CA, USA) supplemented with 2 mM L-glutamine, 10% (V/V) heat-inactivated fetal bovine serum (FBS), penicillin (100 U/ml) and streptomycin (100 μg/ml). The canine B-cell lymphoma cell line CLBL-1 [41] (kind gift of Dr. Barbara Rütgen, University of Veterinary Medicine, Vienna, Austria) was grown in complete RPMI-1640 medium supplemented with 10% (V/V) FBS.

### Generation of rCDVs expressing Venus, dTom and TagBFP fluorescent proteins

Recombinant CDV strain Snyder-Hill (SH) viruses were generated as described previously [10]. Genes encoding the fluorescent reporter proteins Venus, dTom or TagBFP were added as an ATU at the sixth (6) position of the genome (between H and L) (Fig 1A). Importantly, TagBFP and dTom were modified by the addition of six (GGSGSG) and five (GSGSG) amino acids, respectively, to the carboxyl terminus to make them identical in size to Venus (239 amino acids). This ensured that the viral genome lengths were identical from the perspective of replication and transcription by the RNA-dependent RNA polymerase. The three viruses were designated rCDV^SH^Venus(6), rCDV^SH^dTom(6) and rCDV^SH^TagBFP(6). Fluorescence produced in cells infected with these viruses can be readily discerned by flow cytometry, UV epifluorescence microscopy and CLSM. Virus stocks were grown in Vero-cCD150 cells (Fig 1A) and tested negative for contamination with *Mycoplasma* species. Virus titers were determined by endpoint titration in Vero-cCD150 cells and expressed in TCID_50_/ml. Susceptibility of ferret WBC with the reporter viruses was determined *in vitro*. ConA stimulated ferret WBC were inoculated in quadruplicate with rCDV^SH^Venus(6), rCDV^SH^dTom(6) or rCDV^SH^TagBFP(6) at a multiplicity of infection (MOI) of 3 for 1 hour, washed and subsequently cultured for 48 hours. Susceptibility of ferret WBC was analyzed directly by detection of fluorescent reporter proteins by CLSM with a LSM700 system fitted on an Axio Observer Z1 inverted microscope (Zeiss) (Fig 1B) and by flow cytometry on a FACS Canto II (BD Biosciences).

### *In vitro* double infection of CLBL-1 cells

CLBL-1 cells were seeded in 96-well V-bottom plates (Greiner) at 2x10^5^ cells per well. After centrifugation (5 minutes, 350g) supernatants were removed, and cells were resuspended in 150μl culture medium or (combinations of) rCDV(s) diluted to 5x10^4^ TCID_50_ per well in the absence or presence of 10μg/ml of infection-enhancing lipopeptide Pam3CSK4 [65]. After 1 hour at 37°C, the plate was centrifuged, the medium was discarded, and the cells were resuspended in 100μl culture medium (without lipopeptide) and cultured for 23 hours in 96-well flat bottom plates. Infection percentages were determined by flow cytometry (Fig 4D).

In a second experiment, CLBL-1 cells were seeded in 96-well V-bottom plates (Greiner) at 1.2x10^5^ cells per well. After centrifugation (5 minutes, 350g) supernatants were removed, and cells were resuspended in 100μl culture medium or (combinations of) rCDV(s) diluted to 1.2x10^5^ TCID_50_ per well in the absence or presence of 10μg/ml of infection-enhancing lipopeptide PHCSK4 [65]. After 1 hour at 37°C, the plate was centrifuged, the medium was discarded, and the cells were resuspended in 100μl culture medium (without lipopeptide). Five hours later (i.e. six hours after infection 1), cells were centrifuged again and a second infection was performed in the presence or absence of PHCSK4. After 1 hour at 37°C, the plate was centrifuged, the medium was discarded, and the cells were resuspended in 100μl culture medium (without lipopeptide), and cultured for 23 hours in 96-wells flat bottom plates. Infection percentages were determined by flow cytometry (S1 Fig).

### Animal study design

Twelve CDV-seronegative ferrets (*Mustela putorius furo*) were used for the rCDV infection and transmission studies. A temperature probe was implanted intraperitoneally 2 weeks before the beginning of the experiments to monitor body temperature noninvasively. Six donor ferrets (randomly selected, *D1-6*) were inoculated with 10^4^ TCID_50_ rCDV, divided in three equal parts of rCDV^SH^Venus(6): rCDV^SH^dTom(6): rCDV^SH^TagBFP(6) (each 3.3 x 10^3^ TCID_50_). Each reporter virus was administered via a different route, which were alternated over three experiments. Ferrets *D1* and *D2* received rCDV^SH^TagBFP(6) ocularly (Oc), rCDV^SH^dTom(6) intra-nasally (IN) and rCDV^SH^Venus(6) intra-tracheally (IT); ferrets *D3* and *D4* received rCDV^SH^Venus(6) Oc, rCDV^SH^TagBFP(6) IN and rCDV^SH^dTom(6) IT; ferrets *D5* and *D6* received rCDV^SH^dTom(6) Oc, rCDV^SH^Venus(6) IN and rCDV^SH^TagBFP(6) IT (Fig 1C). Oc administration was performed by pipetting virus suspension (50μl) directly onto each of the conjunctivae, IN inoculation by pipetting virus suspension (50μl) into the nostrils while the ferret was held on its back to prevent spread to the trachea and IT inoculations by direct instillation of virus suspension (1ml) into the lower respiratory tract after intubation with a flexible catheter. Following inoculation, the six donor ferrets were placed individually in purpose built cages specifically designed to allow airborne transmission over a 10 cm divide. Donor ferrets were sampled every other day and were euthanized at 14–16 DPI. Six CDV-seronegative recipient ferrets (*R1-6*) were placed in the transmission cages (Fig 6A) at 2 DPI of the donor ferret. Recipient ferrets were sampled every other day and were euthanized at 21/22 DPI (Fig 6B). The animal protocol specified that recipient animals had to be euthanized no later than 22 days after inoculation of the donor animals, which made it impossible to assess the full spectrum of disease in the recipient ferrets.

### Clinical specimens

Small-volume blood samples were collected in Vacuette tubes (Greiner Bio-One, Kremsmünster, Austria) containing K_3_EDTA as an anticoagulant every other DPI of donor ferrets, or every other day after placement of recipient ferrets in transmission cages. Recipient ferrets were always sampled first to prevent direct contamination of recipient animals by sampling of the donor animals. Total WBC and lymphocyte counts were obtained using an automated counter (pocH-100iV; Sysmex). WBC were obtained by lysis of whole blood with red blood cell lysis buffer (Roche, Basel, Switzerland), washed and resuspended in complete RPMI 1640 medium as described above. Cells were counted using a hemocytometer and used directly for flow cytometry and virus isolation. The percentages of WBC infected by different reporter viruses were determined by detection of fluorescent reporter proteins by flow cytometry. Isolation of rCDV was performed on Vero-cCD150 cells using an infectious center test as previously described [66]. Virus isolations were monitored for cytopathic effect (CPE) by microscopy after co-cultivation with Vero-cCD150 cells for 3 to 6 days and results were expressed as the number of virus-infected cells/10^6^ total cells. Relative contribution of the different reporter viruses to the number of virus-infected cells was determined by screening virus isolations on Vero-cCD150 cells for Venus, dTom and TagBFP expression by CLSM.

Throat and rectal swabs (cytobrush plus; Medscand Medical) and nose and eye swabs (polyester-tipped minitip urethral swab; Copan) were collected every other DPI from donor ferrets, or every other day after placement from recipient ferrets, in transport medium (Eagle's minimal essential medium [EMEM] with Hanks' salts, supplemented with lactalbumin enzymatic hydrolysate, penicillin, streptomycin, polymyxin B sulfate, nystatin, gentamicin, and glycerol) and frozen at −80°C. After being thawed, samples were vortexed, the swab was removed, and the remaining transport medium was used for virus isolation. Isolation of rCDV was performed on Vero-cCD150 cells using an infectious center test as previously described [66]. Virus isolations were monitored for CPE by microscopy after co-cultivation with Vero-cCD150 cells for 3 to 7 days and results were expressed as TCID_50_/ml. Relative contribution of the different reporter viruses to the number of virus-infected cells was determined by separately screening virus isolations for Venus, dTom and TagBFP by CLSM.

### Necropsy

Ferrets were euthanized by exsanguination under deep ketamine/medetomidine anesthesia. Macroscopic detection of Venus and dTom was performed with an LED lamp and the appropriate filters as described previously [10, 18]. Post-euthanasia, CSF was obtained by lumbar puncture. Virus isolation from CSF was performed by direct titration on Vero-cCD150 cells. A broncho-alveolar lavage (BAL) was performed postmortem by direct infusion of phosphate-buffered saline (PBS; 5 ml) into the right-hand side of the lung. BAL cells were resuspended in culture medium with supplements as described above, counted, and used directly for flow cytometry and virus isolation. The infection percentages of BAL cells were determined by detection of fluorescent reporter proteins by flow cytometry. Virus isolation was performed on Vero-cCD150 cells as previously described for MV on Vero cells expressing human CD150 [18]. During necropsy, multiple tissues including brain, trachea, primary bronchus, lungs and spleen were harvested and screened directly for expression of fluorescent reporter proteins by CLSM. The left lung was inflated with 2% (W/V) low-melting-point agarose before being screened, as described previously [24, 67]. After screening, non-lymphoid tissues were transferred to 10% neutral-buffered formalin (FA). From two ferrets (*D4* and *D5*), the complete head was stored in 10% neutral-buffered FA for immunohistochemistry. Lymphoid tissues were collected in PBS for preparation of single-cell suspensions using cell strainers with a 100 μm pore size (BD Biosciences, Erembodegem, Belgium) and directly used for flow cytometry. The infection percentages of single cell suspensions by different reporter viruses were determined by flow cytometry.

### Histological and immunohistochemical analysis

After fixation in 10% formalin, ferret heads were decalcified in 10% EDTA (pH 7.4) for at least a month. After decalcification heads were embedded in paraffin. CDV^SH^ was detected using a monoclonal antibody (VMRD Inc., Pullman, WA, USA). Briefly, 3μm paraffin sections were deparaffinized and antigens were retrieved by boiling slides for 15 minutes in citric acid buffer (10mM, pH 6.0). Sections were incubated with the anti-CDV antibody for 1 hour at RT. Binding of the primary antibody was detected using a biotinylated rabbit-anti-mouse Ig (DAKO), after which tissue sections were incubated with ABComplex-HRP (DAKO) for 30 minutes. Peroxidase was revealed using 3-Amino-9-ethyl-carbazole (AEC, Sigma) resulting in a bright red precipitate. In each staining procedure an isotype control was included as a negative control.

## Supporting information

S1 Fig *In vitro* single and double infections of canine B-lymphoblastoid cell line CLBL-1. (A) Experimental design: CLBL-1 cells were infected at T = 0 and / or T = 6 hours. One hour after the infections (or incubation with culture medium as mock control), cells were washed to remove unbound virus. At T = 30 hours infection percentages were determined by flow cytometry. (B) List of the conditions (#) applied for infection 1 (I_1_) or 2 (I_2_). All infections were performed in triplicate, using a multiplicity of infection of 1, in the presence or absence of the infection-enhancing lipopeptide PHCSK4 [63]. (C) Infection percentages (irrespective of which reporter protein was expressed) ranged from 14.5 to 51.3% or from 64.1 to 84.8% when performed in the absence (top) or presence (bottom) of infection-enhancing lipopeptide, respectively. (D) Examples of confocal scanning laser microscopy (top) or flow cytometry (bottom) measurements of conditions 1 (mock), 2 (Venus), 3 (dTom) and 6 (Venus + dTomOM) 30 hours post-infection in the presence of PHCSK4. In the FACS plots, single positive cells are shown in green (Venus) or red (dTom), while double-positive cells are shown in yellow. (E) Distribution of single- or double-positive cells in the infected cell populations of conditions 2–8.
(TIF)

S2 Fig rCDV was detected in the central nervous system (CNS) and nasal cavity of directly inoculated ferret *D5*. Immunohistochemistry for the detection of CDV was performed on (A) complete ferret head sections (red box in inset shows anatomic location of section) and showed rCDV presence in the CNS (cerebellum [Cl], cerebrum and olfactory bulb). (B) CDV-positive cells within the ependyma and choroid plexus (arrow). (C) CDV was detected in endothelial cells and cells surrounding the blood vessel, suggestive of a hematogenous spread of rCDV to the brain. (D) CDV-positive cells were detected in neurons and glial cells of the olfactory bulb. (E) CDV-positive neurons and glial cells in the cerebrum.
(TIF)
