# Supplementary figures and images for: Delineating morbillivirus entry, dissemination and airborne transmission by studying in vivo competition of multicolor canine distemper viruses in ferrets
Source: PLoS Pathog. 2017 May 8;13(5):e1006371. doi: 10.1371/journal.ppat.1006371 (PMC5436898; doi:10.1371/journal.ppat.1006371)

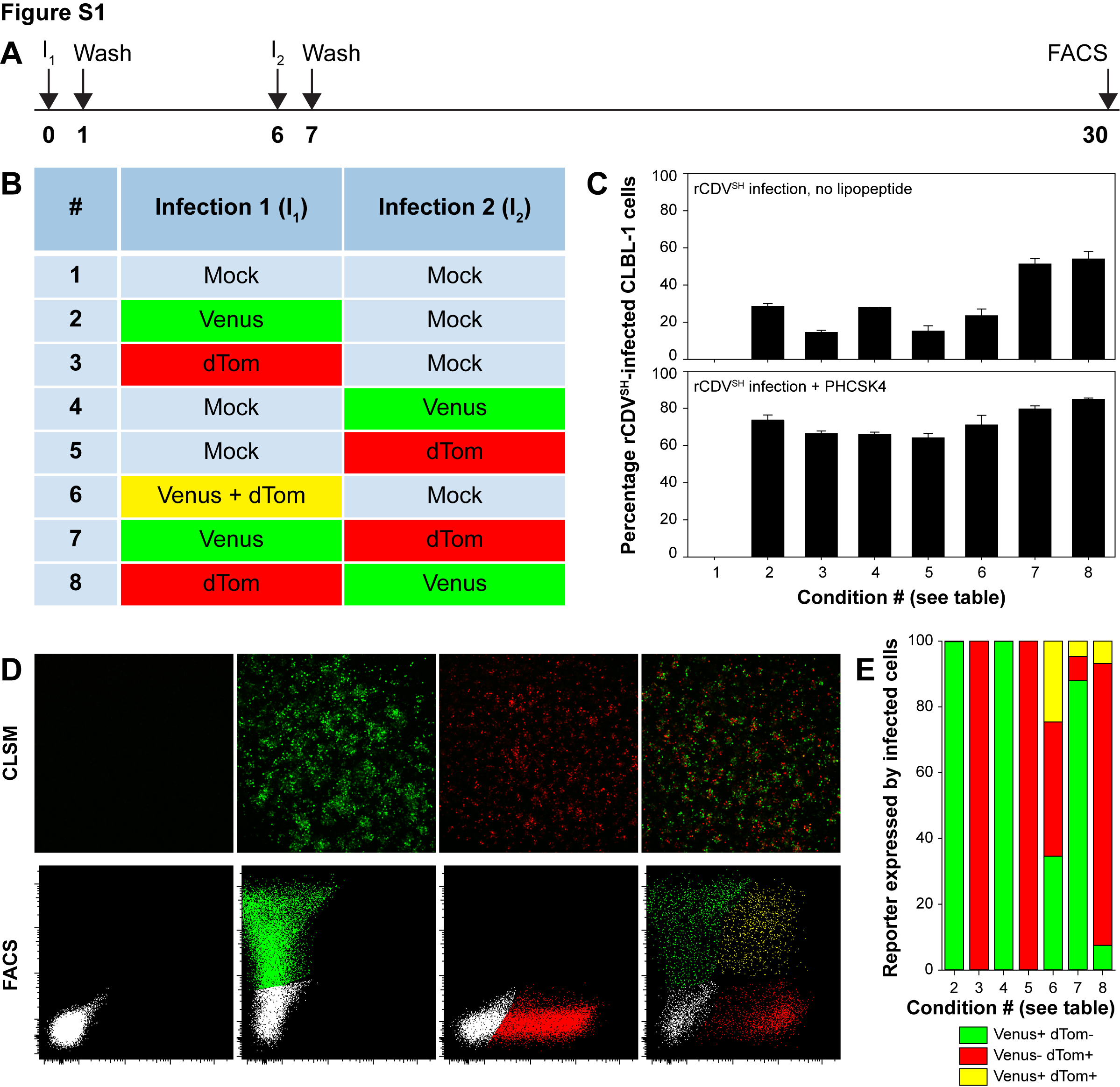

Supplement: S1 Fig — (A) Experimental design: CLBL-1 cells were infected at T = 0 and / or T = 6 hours. One hour after the infections (or incubation with culture medium as mock control), cells were washed to remove unbound virus. At T = 30 hours infection percentages were determined by flow cytometry. (B) List of the conditions (#) applied for infection 1 (I1) or 2 (I2). All infections were performed in triplicate, using a multiplicity of infection of 1, in the presence or absence of the infection-enhancing lipopeptide PHCSK4 [63]. (C) Infection percentages (irrespective of which reporter protein was expressed) ranged from 14.5 to 51.3% or from 64.1 to 84.8% when performed in the absence (top) or presence (bottom) of infection-enhancing lipopeptide, respectively. (D) Examples of confocal scanning laser microscopy (top) or flow cytometry (bottom) measurements of conditions 1 (mock), 2 (Venus), 3 (dTom) and 6 (Venus + dTomOM) 30 hours post-infection in the presence of PHCSK4. In the FACS plots, single positive cells are shown in green (Venus) or red (dTom), while double-positive cells are shown in yellow. (E) Distribution of single- or double-positive cells in the infected cell populations of conditions 2–8. (TIF) [file ppat.1006371.s001.tif]

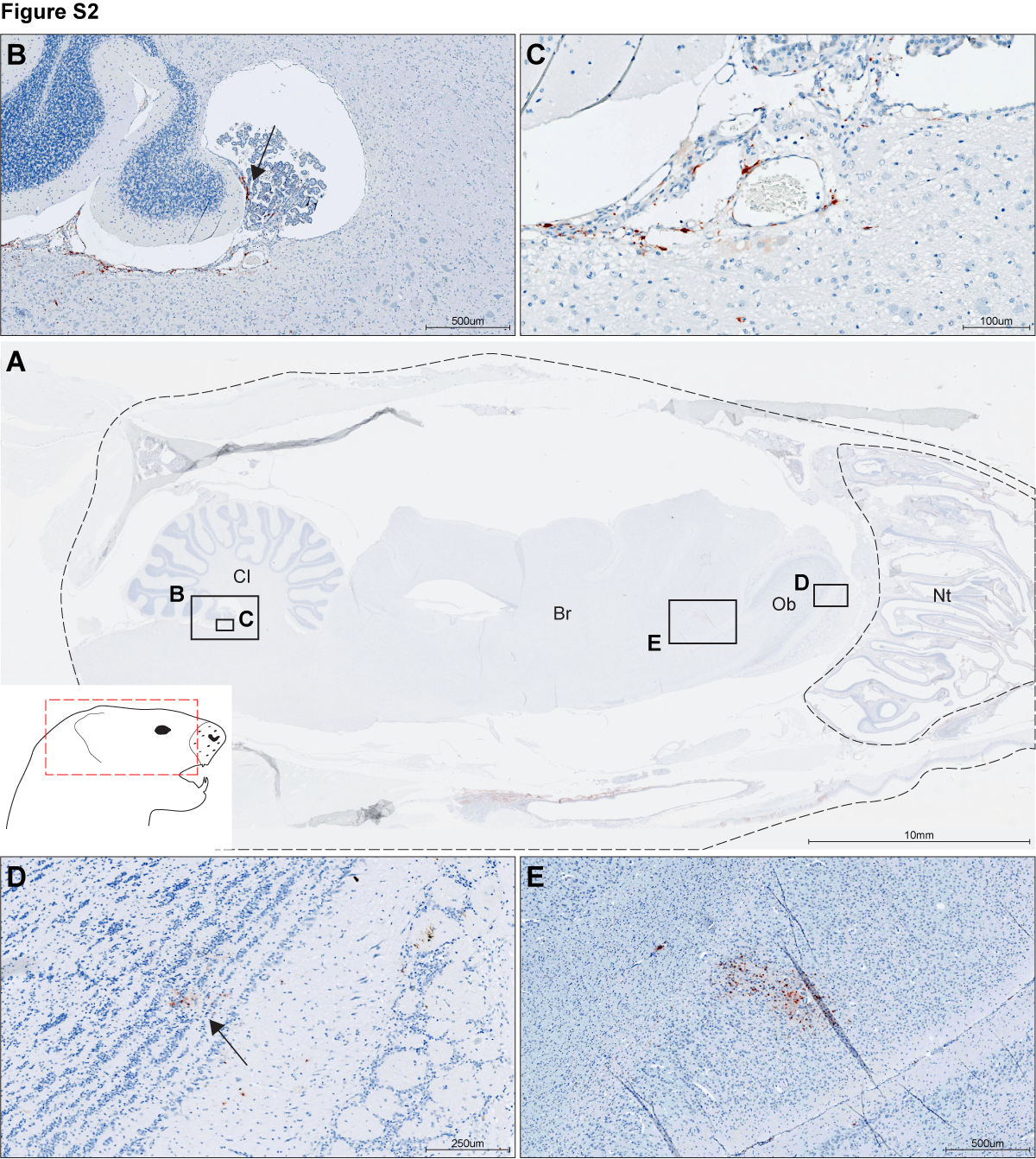

Supplement: S2 Fig — Immunohistochemistry for the detection of CDV was performed on (A) complete ferret head sections (red box in inset shows anatomic location of section) and showed rCDV presence in the CNS (cerebellum [Cl], cerebrum and olfactory bulb). (B) CDV-positive cells within the ependyma and choroid plexus (arrow). (C) CDV was detected in endothelial cells and cells surrounding the blood vessel, suggestive of a hematogenous spread of rCDV to the brain. (D) CDV-positive cells were detected in neurons and glial cells of the olfactory bulb. (E) CDV-positive neurons and glial cells in the cerebrum. (TIF) [file ppat.1006371.s002.tif]
